# Supplementary material for: Co-Existence of Atomic Pt and CoPt Nanoclusters on Co/SnOx Mix-Oxide Demonstrates an Ultra-High-Performance Oxygen Reduction Reaction Activity
Source: Nanomaterials (Basel). 2022 Aug 17;12(16):2824. doi: 10.3390/nano12162824 (PMC9413684; doi:10.3390/nano12162824)
Supplement: Supplementary file 1 [file nanomaterials-12-02824-s001.zip › nanomaterials-1865379-supplementary.pdf]

Supplementary Materials

Co-Existence of Atomic Pt and CoPt Nanoclusters on Co/SnO$_x$ Mix-Oxide Demonstrates an Ultra-High-Performance Oxygen Reduction Reaction Activity

Amisha Beniwal [1], Dinesh Bhalothia [1], Wei Yeh [1], Mingxing Cheng [1], Che Yan [1], Po-Chun Chen [2], Kuan-Wen Wang [3] and Tsan-Yao Chen [1,4,*]

[1] Department of Engineering and System Science, National Tsing Hua University, Hsinchu 30013, Taiwan
[2] Department of Materials and Mineral Resources Engineering, National Taipei University of Technology, Taipei 10608, Taiwan
[3] Institute of Materials Science and Engineering, National Central University, Taoyuan City 32001, Taiwan
[4] Hierarchical Green-Energy Materials (Hi-GEM) Research Centre, National Cheng Kung University, Tainan 70101, Taiwan
* Correspondence: chencaeser@gmail.com; Tel.: +886-3-5715131 (ext. 34271); Fax: +886-3-5720724

1. **ICP-OES results of CSPP NC.**

Table S1. The ICP-OES determined actual composition of CSPP NCs.

Sample	Composition (%)			
	Co	Sn	Pd	Pt
CSPP-RT/50/70	2.747	6.095	5.472	0.329

- The composition is same for all the CSPP NCs.

2. HRTEM Image of CSP Nanocatalyst.

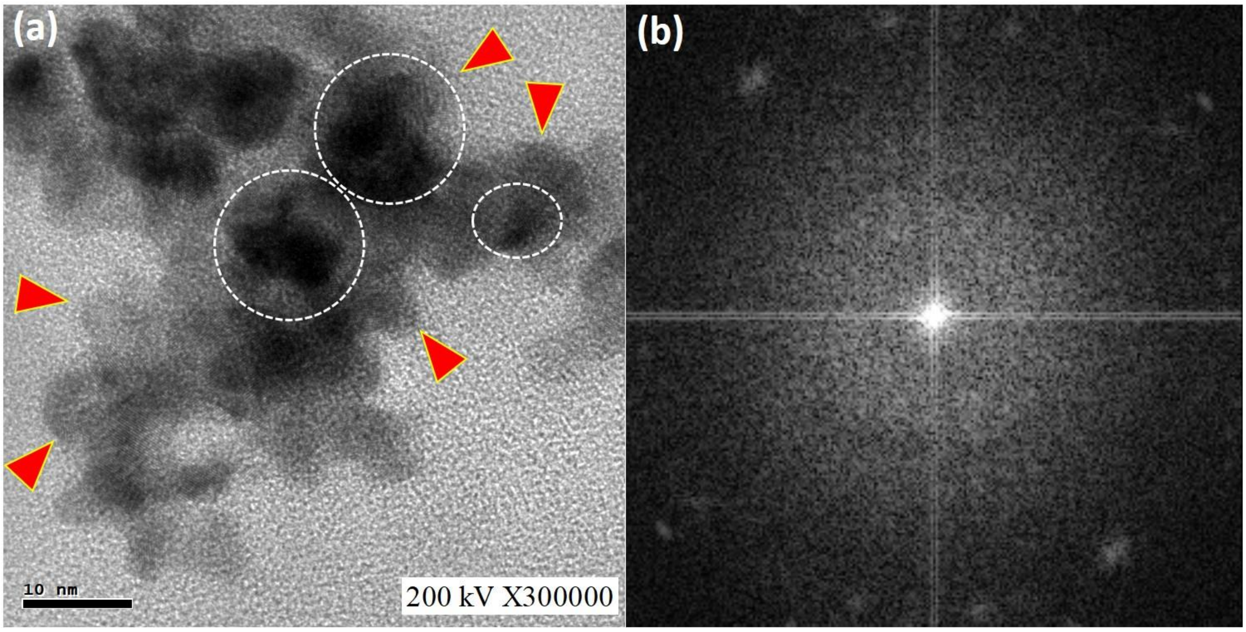

Figure S1. (a) The HRTEM image and (b) corresponding FFT pattern of CSP nanocatalyst.

3. The XRD Patterns of control samples.

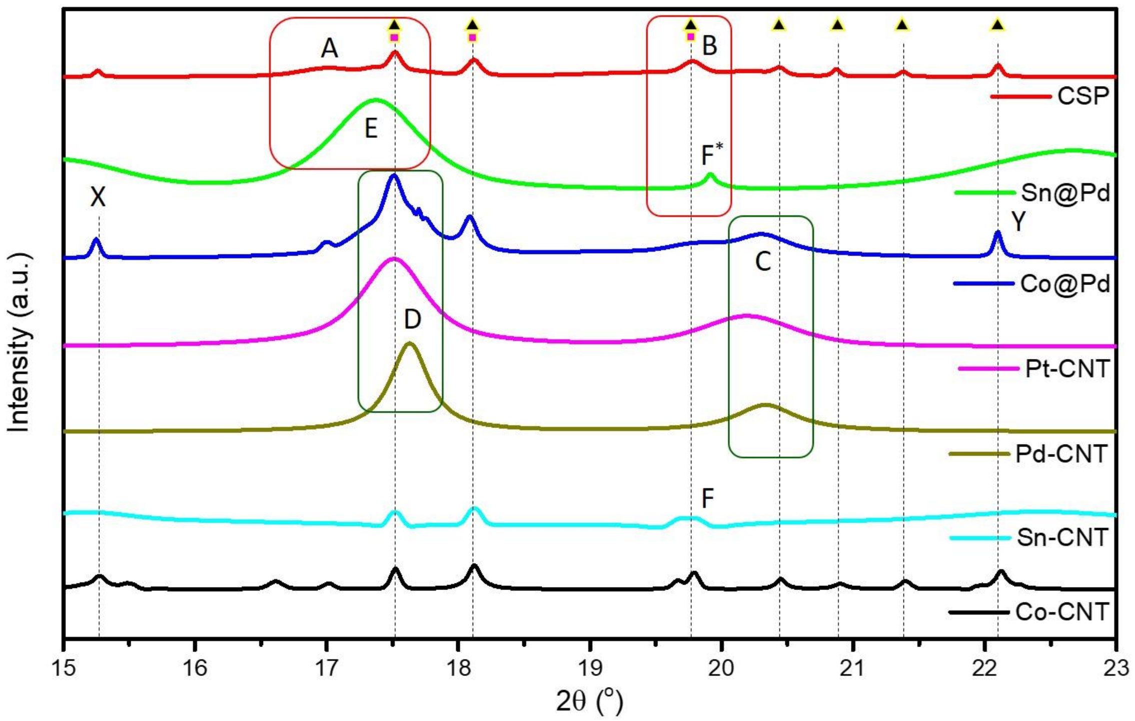

Figure S2. The XRD patterns of control samples.

Table S2. XRD determined average particle size of experimental NCs.

Sample	Component	2θ position (°)	FWHM	Average size (nm)
Pd-CNT	Pd (111)	17.63	0.511	6.94
Pt-CNT	Pt (111)	17.50	0.864	4.11
Sn@Pd	SnPd alloy	17.40	1.040	3.41
CSPP-RT	SnO_2 phase	17.53	0.072	47.56
CSPP-50	CoPt (110)	20.87	0.070	50.96
	CoPt (002)	21.05	0.090	39.65
	SnO_2 phase	17.49	0.084	42.26
CSPP-70	SnO_2 phase	15.54	0.071	47.96

4. Electrochemical parameters of experimental NCs

Table S3. Electrochemical parameters of experimental NCs

Sample	N (0.5 V)	ECSA ($cm^2\ mg_{Pd+Pt}^{-1}$)	V_{oc} (V)	$E_{1/2}$ (V)	J_K^o (0.85 V)	J_K^c (0.85 V)	M.A. $_{Pt/Pd\ (0.85V)}$ (mA/mg)	S.A.$_{(0.85V)}$ ($mA\ cm^{-2}$)
CSP	3.24		0.896	0.834	1.95	1.95	36.30	
CSPP-RT	4.39	111.6	0.913	0.867	5.23	3.28	1669	0.523
CSPP-50	3.85	110	0.946	0.894	10.46	8.51	4330	1.045
CSPP-75	3.21	97.7	0.860	0.810	1.55	N/A	N/A	0.219

* For CSPP NCs, J_K^c $_{(0.85\ V)}$ is calculated by deducing the contribution of the J_K^o $_{(0.85\ V)}$ of CSP from that of CSPP NCs. The J_K^c $_{(0.85\ V)}$ is used for calculating the mass activities of experimental NCs.

5. Electrochemical results of CSPP NCs compared with CSP NC

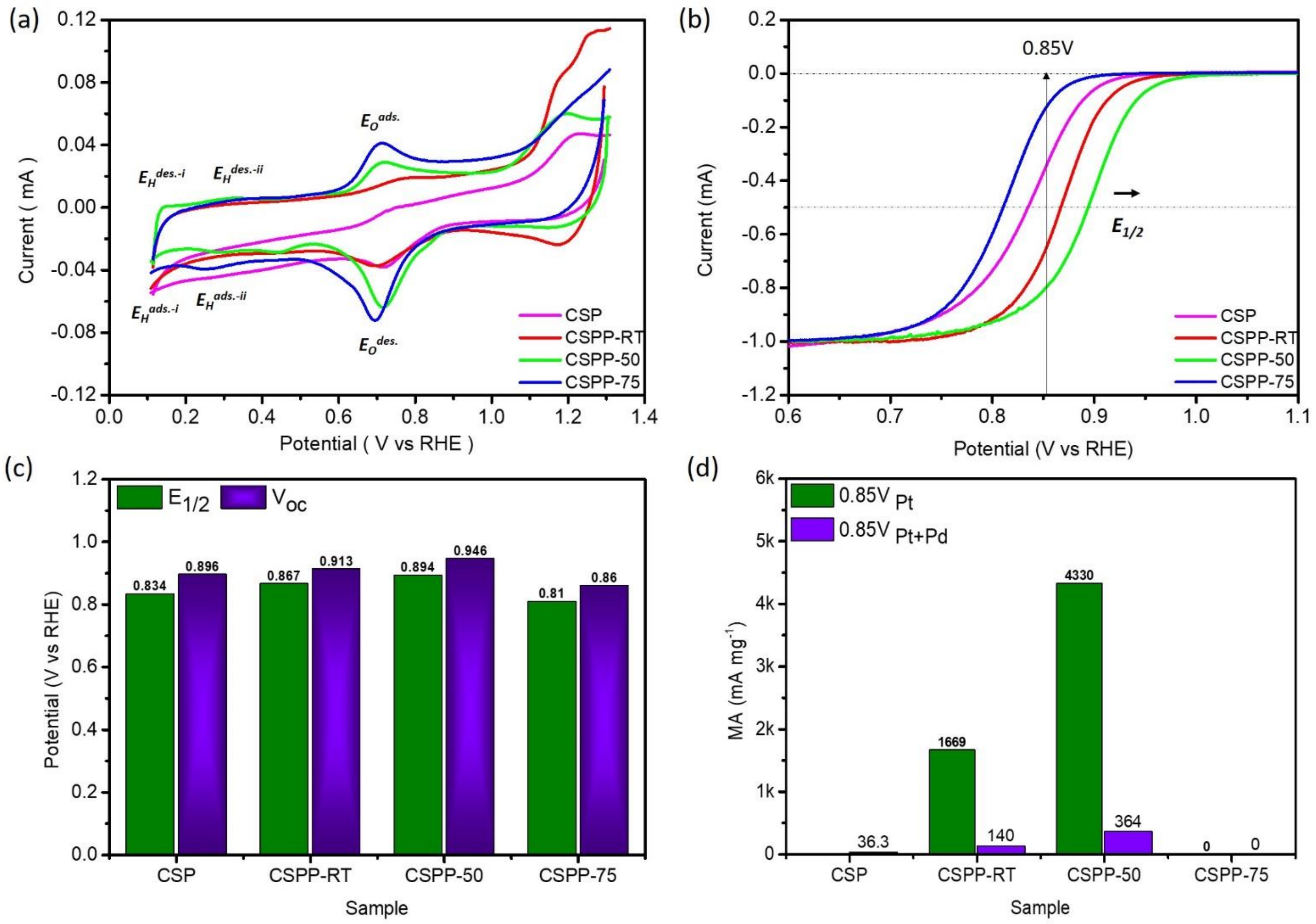

Figure S3. Electrochemical results of CSPP NCs compared with CSP NC. (a) CV and (b) LSV curves of CSPP-RT, CSPP-50 and CSPP-75 NCs compared with reference sample (CSP). (c) Onset potential (Voc)/half wave potential ($E_{1/2}$) and (d) ORR mass activity of CSPP NCs compared with commercial J.M.-Pt/C NCs.
